# Discursive Marketisation Through Positive Evaluation: A Diachronic Analysis of *About Us* Texts of Top-Tier Chinese Universities Over the Past Two Decades

**DOI:** 10.3389/fpsyg.2021.789558

**Published:** 2021-12-02

**Authors:** Lihua Xu, Chaoqun Xie, Jun Lei

**Affiliations:** ^1^School of Foreign Languages, Neijiang Normal University, Neijiang, China; ^2^Language Academy, Universiti Teknologi Malaysia, Johor Bahru, Malaysia; ^3^Faculty of Foreign Languages, Ningbo University, Ningbo, China

**Keywords:** Chinese universities, marketisation, diachronic analysis, attitude, appraisal

## Abstract

This study explored the marketisation process of top-tier Chinese universities by scrutinising their self-promotional strategies over the past two decades. Drawing on Martin and White’s (2005) Appraisal framework, we identified all attitudinal markers in the *About Us* texts posted by 35 top-tier Chinese universities on their official websites at two time-points: the turn of the century and the year of 2021. The 35 universities were drawn from China’s “Double First Class” Initiative that prioritises the development of a select group of elite universities in China. Close textual analyses focussing on the attitudinal markers with reference to their contexts were conducted to identify the themes evaluated in the *About Us* texts; Wilcoxon signed-rank tests were run to quantitatively compare the relative frequencies of the attitudinal markers between the two phases, which was then supplemented by diachronic qualitative comparisons on the fine-grained linguistic features surrounding the markers. The study identified seven major themes positively appraised by the universities at both time-points. It also revealed diachronic differences in the use of attitudinal markers, reflecting a mediated change of promotional strategies over the past 20 years or so in the Chinese higher education context. These findings point to the influence of market, government, and tradition on Chinese top-tier universities’ promoting strategies and the role of social cognition in shaping student choice. They also suggest the emergence of a higher education system with Chinese characteristics that features a reconciliation of market and government forces.

## Introduction

The powerful encroachment of globalisation and neoliberalism has accelerated the marketisation of higher educational institutions worldwide. As a result, universities are increasingly seen as competing entities that endeavour to commercialise their educational products in the global higher education marketplace. To commodify education, marketised universities adopt managerial protocols originally prevalent in the business world, such as quality control, auditing and ranking performance, quantifying students’ experience, and vying for a favourable position in league tables (Furedi, 2011; Molesworth et al., 2011).

This marketisation logic is also manifest in Chinese universities, as they are observed to lure quality faculty with attractive monetary incentives (Feng, 2019), build university-run enterprises to generate revenues (Eun et al., 2006), set publishing requirements and rewards schemes for faculty promotion and doctoral students’ graduation (Lei, 2021). However, the marketisation of Chinese universities has its own characteristics due to their unique social milieu and functions. The backbone of Chinese higher education are state-owned public universities, which means that institutional fundings, appointment of university leadership, quotas of faculty and student recruitment are predominately determined by the government, most frequently the Chinese Ministry of Education (MOE). In other words, the marketisation process of Chinese universities is guided and supervised by the government. This mediated version of marketisation has been noted as a strong contributing factor not only for succumbing challenges posed by the prevailing neoliberalism, but also for gradually bringing Chinese prestigious universities to the forefront in the global higher education sector. While only five universities in Chinese mainland entered the Times Higher Education Top 200 World universities Ranking in 2004, the number doubled in the same ranking in 2021 (Times Higher Education, 2021).

Top-tier universities in China have been generously funded by the central government since the 1990s. Different supporting strategies and initiatives such as the Project 211, Project 985 and Double First Class Initiative have provided continuous financial and policy support to foster the development of top-tier Chinese universities in different historical periods. The Double First Class Initiative, the latest national strategy, aims to help dozens of Chinese universities become influential competitors in the global higher education marketplace.

Owing to its convenient and economic accessibility, the Internet has served as an increasingly popular tool for universities’ self-promotional activities over the past 20 years or so. Chinese universities, like their Western counterparts, have capitalised on their official websites to publicise their strengths and features so as to promote their brands and attract prospective students. This study focuses on the texts on the *About Us* webpages of top-tier Chinese universities, and investigates how the promotional strategies, and more broadly the marketisation process, are verbally reflected in the virtual genre over the past two decades.

## Study Background

### University and Self-Promotion

Self-promotion has become all the more popular among universities around the world as it has grown increasingly competitive for institutions of higher learning to attract quality faculty and students, obtain public and private funds, and secure collaboration with industries. Universities have deployed a variety of institutional activities to carry out self-promotion. Open Days, for example, provide an opportunity for the universities to invite perspective students and their parents to visit their campuses to get to know more about various aspects of the universities through live experience and engagement (Wernick, 1991). Students’ brochures and prospectuses are distributed to promulgate the universities, and newspaper advertisements are posted to attract high-profile faculty members to join the institutional enterprise (Fairclough, 1993; Xiong, 2012). In this Internet age, social media and institutional official websites are also useful platforms for self-promotional purposes (Feng, 2019; Zhang and Tu, 2019; Zhang et al., 2020).

In all these promotional activities semiotic symbols play a vital role, as the success and efficiency of these self-propagandas depend heavily on the way in which the universities are visually or verbally presented to their stakeholders. Visual semiotics, due to the special affordances they possess, can be communicated to the audience in a more straightforward manner (Martinec and Salway, 2005; Bateman, 2014). As a result, universities often present visual images that can evoke positive evaluation from the audience to achieve their promotional goals. For example, they often show in their enrolment brochures successful and happy students wearing graduation gowns, capable teachers giving specialised instructions, researchers demonstrating their latest inventions, picturesque campus sceneries and landscapes, and advanced infrastructure and facilities. In addition to the content of the images, they also strategically calibrate the ways in which the images are presented (Kress and van Leeuwen, 2006). When featuring individuals, they often choose close shots rather than middle or longshots. Direct or oblique shots are selected to create desired promotional effects. Low angle pictures are preferred over high angle ones when university architectures are presented, because the former tend to create an imposing and remarkable impression and make an authoritative and noble university image (Tu, 2016). The colour and design of university websites seem to have multiple promotional functions, including interpersonally denoting the institutional identity, directing visitors’ attention, and creating a welcome feeling and atmosphere (Zhang and O’Halloran, 2013).

Studies have also probed the use of symbolic meaning and visual metaphor in pictures presented by the universities (Ng and Koller, 2013; Ng, 2016). Ng and Koller (2013), for example, found that positive values (e.g., freedom, flexibility, and empowerment) were visually communicated by foregrounding symbolic representations, which were frequently expressed through decontextualisation (e.g., without clear verbal anchoring) and improbable content that subverted naturalistic norms and expectations (e.g., students tossing the dean up in the air). Moreover, it was found that visual metaphors in universities’ prospectuses endowed inanimate entities with human cognition and emotion, which represented the university favourably and thereby enhanced its branding effects on its potential students (Ng, 2016).

Aside from visual pictures, some research has focussed on the emblem and logo of universities and investigated the promotional functions performed by these visual elements. Symbolic meanings represented in universities’ icons have been found to contribute to visualising institutional identities–corporates competing in the higher education market or organisations enacting local and academic tradition, contextualised in specific cultures (Drori et al., 2016; Oberg et al., 2017). Such identity construction and presentation work to tell stories about the universities, appealing prospective students to be part of the ongoing institutional narratives.

Compared with the visual semiotics, the promotional mission executed by verbal texts has drawn much more research attention. Texts in students’ brochures and prospectuses, faculty recruitment advertisements, missions and visions, university self-introductions, presidents’ welcoming messages and graduation speeches have been extensively analysed with varying theoretical underpinnings and analytic lenses. Some researchers took a genre perspective and decoded the rhetorical moves of the verbal texts, based on which the promotional agenda behind the moves was revealed (Xiong, 2012; Han, 2014). Others focussed on types of discourse that have been appropriated in the higher education contexts, such as tourism discourse, business discourse, advertisement discourse, narrative discourse, and policy discourse. Interdiscursivity constructed by integrating and hybridising these types of discourse increased the complexity of communicative purposes, reflecting the growing promotional culture in the higher education context (Fairclough, 1993; Michelson and Valencia, 2016; Feng, 2019; Teo and Ren, 2019). Teo and Ren (2019), for example, examined the rhetorical structure and discursive strategies in presidents’ messages published on official websites of 36 top-ranked universities in China. A dynamic interweaving of three discursive strands were identified: bureaucratic, conversational and advertising discourses. The co-existence and intermix of the three types of discourse, as discussed by the authors, revealed the dual role of the presidents’ messages: to project an international outlook of the Chinese universities and to maintain their allegiance to dominant political ideologies and national interests.

Still other researchers have zoomed in on the lexico-grammatical resources in the texts from the perspectives of corpus linguistics and/or systemic functional linguistics (SFL). For example, adopting the corpus-based approach, Mautner (2005) revealed that there was a prevalence of keywords with the word root of *enterprise* (e.g., *entrepreneurship* and *entrepreneurial*) in the texts on universities’ websites, clearly suggesting the commercialisation of higher education. The study also found that the universities tended to promote their academic entrepreneurship through ample use of positively loaded lexis on both sides of the searched keywords. Another corpus study on university websites revealed that the keyword *research* was often collocated with *quality* and *world*, indicating that quality and globalisation of research were foregrounded in the self-promotion (Sanigar, 2013). Moreover, drawing on the systemic functional linguistics (SFL) framework as an analytic tool, research (e.g., Askehave, 2007; Teo, 2007) has scrutinised ideational, interpersonal and textual meanings construed in universities’ promotional texts. This strand of research has highlighted the role of interpersonal meaning in the creation of positive institutional image and the establishment of desired institution-audience relationship through deliberate manipulation of various linguistic devices. Stemming from the interpersonal dimension, the concept of evaluation has taken the centre stage in the construction of *style*, the way institutional or personal identities are discursively constructed (Fairclough, 2003). In this regard, Appraisal theory developed by Martin and White (2005) is regarded as a theoretically grounded and systematically delineated conceptualisation of evaluative meanings (Hood, 2004; Hunston, 2011; Alba-Juez and Thompson, 2014).

### Appraisal and Attitude

According to Martin and White (2005), Appraisal deals with evaluative resources concerning people’s feelings (i.e., Attitude), dialogistic value positioning (i.e., Engagement), and grading phenomena whereby feelings and positioning are adjusted in written or spoken locutions (i.e., Graduation). As part of a larger research project, this study focuses solely on the Attitude dimension. Attitude concerns people’s inner feelings and emotions (e.g., happiness, satisfaction, desire, and security) (i.e., Affect), judgements of people’s characters and behaviours (e.g., capacity, bravery, diligence, propriety, honesty) (i.e., Judgement), and evaluation of things and abstract qualities (e.g., value, attractiveness, and loftiness) (i.e., Appreciation).

Previous studies have employed the Attitude framework to investigate the evaluative meanings communicated in universities’ promotional texts (Morrish and Sauntson, 2013; Xie and Teo, 2020a). It is noteworthy that these studies tend to take a comparative lens. For example, Morrish and Sauntson (2013) compared how the older, research-intensive universities and the younger business-oriented universities used attitudinal resources to fulfil their promotional goals, and found that the former group of universities mostly appraised *themselves* in a positive manner, whereas the latter group evaluated entities external to them, such as local industry and technology. Xie and Teo (2020a) compared the use of Attitude markers in the *About Us* texts of top-tier and second-tier universities in China and America. The examination of the Attitude markers revealed that while Chinese universities seemed to construct a more university-centric, authoritative, and distant image, their American counterparts tended to build a more people-oriented, friendly, and approachable image. The top-tier universities in both countries underscored their prestige and history, while the second-tier ones appeared to foreground the positive college experience their students could enjoy.

Apart from the cross-country and cross-tier comparisons, there were also diachronic studies that compared the promotional texts produced at different historical periods, though this research did not focus specifically on Attitude markers (e.g., Fairclough, 1993; Zhang and O’Halloran, 2013). These studies uncovered a historical shift in the objectives and functions of promotional texts shaped by the broader social setting of accelerated marketisation of higher education: a progressive transition from communicating ideational information to tactically manipulating the information for promotional purposes.

There is no doubt that the studies reviewed above have shed much light on the marketisation of higher education. However, what seem to be under-researched are how this historical transition is discursively manifested, and how Attitude markers contribute to the realisation of such discursive manifestation. In view of these gaps, the following research questions are posed to guide this study.

1. What are the major themes positively evaluated in the top-tier Chinese universities’ *About Us* texts?
2. Are there changes in the use of attitudinal markers in the *About Us* texts over the past two decades?
3. What meaning construction has been realised in the *About Us* texts over the past two decades?

## Materials and Methods

### Research Design

This study employed a mixed method research design to answer the three research questions. Detailed lexico-grammatical analysis of the *About Us* texts was carried out with reference to the contexts to locate Attitude markers and to identify themes related to the markers. Statistical measures and close textual examination were employed to address the second research question: the diachronic comparisons on the use of the markers. To these ends, an *About Us* corpus was constructed to cater to the fine-grained lexico-grammatical, thematic, and statistical analyses. Finally, the themes identified in the first research question and the diachronic changes noted in the second one were brought together and scrutinised to answer the third research question.

### Data Collection

To answer the three research questions, we collected online *About Us* texts of the selected Chinese universities as our raw materials. The collection of data involved the following steps: sampling top-tier Chinese universities, retrieving the online *About Us* texts of the sampled universities at the two time points, and importing the raw texts into a corpus tool for the ensuing coding and analysis of attitude markers.

As our study was interested in the attitudinal markers in the *About Us* texts of top-tier Chinese universities, we selected the very best universities listed in the ‘‘Double First Class’’ universities (Category A) by the Chinese MOE, which included 36 prestigious universities in Mainland China. While it posed little challenge to retrieve the latest *About Us* texts posted on their official webpages by the 36 universities, we initially had some difficulties in getting the earlier versions of *About Us* texts posted by these universities around 2,000, because the websites had been regularly or irregularly updated, and the texts had kept changing. The issue was finally addressed by the Wayback Machine^1^, an Internet archival library, which could help get the earlier versions of the online texts of 35 universities, with the earliest being East China Normal University (January, 1997) and the latest Fudan University (May, 2004). As a result, these 35 universities were sampled for this study and correspondingly their *About Us* texts posted around 2000 (Phase I) and 2021 (Phase II) were retrieved. Table 1 shows the total number of words and the average number of words per text for the two phases.

**TABLE 1:** Descriptive statistics for the *About Us* corpus.

**Year**	**Total number of words**	**Average number of words per text**
Phase I (around 2000)	23,251	664.31
Phase II (2021)	29,800	851.43

### Data Coding

The 70 texts were then imported into a corpus tool, UAM CorpusTool, for coding and further analysis. The Attitude Framework, as the coding scheme, was inputted into the CorpusTool to facilitate the coding process. Both automated and manual coding approaches were used to assign coding labels of the coding scheme to the text segments with any attitudinal meanings. A second coder who was familiar with the Appraisal framework and the operation of the UAM CorpusTool was invited to code 10% of the data. Her coding results were compared against with the results coded by the first author, and a satisfactory inter-coding agreement (94.3%) was observed. Fuzzy and ambiguous cases were settled through discussion and consulting with an expert in discourse semantics and a senior administrator of a university. Subsequent to these steps, the first author coded all the remaining data. Coding validity was also checked through two rounds of reviewing and within-group comparison among instances assigned with the same labels.

### Data Analysis

Data analysis in this study involved both textual examination which was qualitative in nature and statistical comparisons which were quantitative in nature. For the first research question, instances of the different types of Attitude markers identified through the CorpusTool were closely examined to explore the themes positively evaluated in the *About Us* texts. Particular attention was paid to the contents and the contexts of the Attitude instances, such as the appraiser/appraised, the polarity of the Attitude construed, and the characteristics displayed in the evaluations.

As for the second research question, statistical analysis was first conducted to compare the frequencies of different types of Attitude at the two time points. To offset the text length effects, we standardised the raw frequencies of attitude markers in the corpora to relative frequencies (per 1,000 words) for the diachronic comparisons. Both Kolmogorov–Smirnov and Shapiro–Wilk tests showed that the standardised data from the Phase I corpus were not normally distributed. As such, we ran four Wilcoxon signed-rank tests to compare the relative frequencies of the four types of markers (for Attitude, Affect, Judgement, and Appreciation) between the two phases. Wilcoxon signed-rank tests are non-parametric tests where two sets of data from the same participants are compared when normality in the data is not assumed. The α value was set at 0.05 for all the Wilcoxon signed-rank tests. The statistical tests were then followed by nuanced qualitative analysis, where instances of the different types of Attitude markers were scrutinised to identify diachronic differences in construing the attitudinal meanings. The UAM CorpusTool automatically categorised the coded textual extracts into different groups according to their attitudinal meanings, so that grouped examples were offered for detailed textual analysis. In-depth textual analysis facilitated a contextualised understanding of the attitudinal markers. As with the theme analysis noted above, we also considered the contents and the contexts of the Attitude instances in the in-depth textual analysis.

The analysis of data for the last research question was primarily based on the answers to the first two research questions. The major themes unveiled and the diachronic differences identified in the earlier analyses were compared and synthesised to explore the underlying meanings constructed in the texts.

## Findings

Our analysis of the corpus data revealed a high density of attitude markers in the *About Us* texts. Altogether, 2,598 Attitude markers were identified in the two corpora, 925 in the Phase I corpus and 1,673 in the Phase II corpus. Table 2 shows the breakdown distribution of these markers across the three dimensions of Attitude. Among these dimensions, a consistent distribution pattern can be observed: the Appreciation markers had the highest occurrence, followed in order by the Judgement markers and the Affect markers. In terms of polarity, the overwhelming majority of the Attitude markers identified were positive evaluations, with only a few (*n* = 6) negative instances.

**TABLE 2:** Attitude in the *About Us* texts: Means (standard deviations).

	**Phase I Corpus (around 2000)**	**Phase II Corpus (2021)**
Attitude	39.43 (3.20)	52.64 (2.99)
Affect	0.34 (0.17)	0.29 (0.12)
Judgement	7.57 (1.06)	11.28 (1.34)
Appreciation	31.52 (2.91)	41.07 (2.97)

### Major Themes Positively Evaluated

Our detailed textual analysis revealed seven major themes positively evaluated in the *About Us* texts of the selected top-tier Chinese universities during the past two decades, including geographic location/city, scenery and landscape, history and culture, academic and research strength, faculty capacity, alumni and students, and international collaboration and cooperation.

#### Geographic Location/City

Given the fact that all the top-tier universities are located in metropolitans with glamorous social resources, rich cultural heritage and abundant employment opportunities, the universities made full use of such advantages to attract prospective students. Extracts 1 and 2 are examples where the host cities are positively evaluated for the institutions’ promotional purposes.

(1) HUST is located in Wuhan, a **famous** city in the middle reaches of the Changjiang (Yangtze River) (Huazhong University of Science and Technology, 1997).
(2) Located in the city of Guangzhou, a **thriving** metropolis in South China, the university today covers a total area of 391 hectares (South China University of Technology, 2021).

#### Scenery and Landscape

The universities often prided themselves on their scenery and environment, which were always portrayed as attractive and pleasant. Such portrayals and evaluations were generally arranged in the texts prior to the promulgation of academic matters, the signature feature of higher education. This indicates universities’ strategy of prioritising a cosy and enjoyable learning environment over learning *per se*. Extracts 3 and 4 are overt examples where campus scenery and landscape are positively appraised.

(3) The university is set amidst **picturesque** scenery, with its campus running along the seashore at the foot of mountains (Xiamen University, 1996).
(4) The university campuses, with their **favourable** environment and **beautiful** landscape, make a wonderful place for learning and research (Sichuan University, 2021).

#### History and Culture

Aside from the concrete and tangible physical entities, soft powers of universities, such as institutional history, tradition, culture and spirit, were also accoladed, as seen in Extracts 5 and 6.

(5) Wuhan University is a famous university with a **long** history, a **glorious** revolutionary tradition and a **fine** academic tradition (Wuhan University, 1998).
(6) SEU, as one of the **time-honoured** institutions of higher learning in China with **profound** cultural heritage, its origin can be traced back to 1902 (Southeast University, 2021).

#### Academic and Research Strength

Although the aforementioned themes are instrumental in attracting prospective students, what students care most about in the universities are learning opportunities, acquisition of academic knowledge, and development of abilities. In this regard, these top-tier universities have a competitive edge over less prestigious universities, and thus made frequent positive evaluations in relation to their strengths in academic disciplines and research outputs, as exemplified in Extracts 7 and 8.

(7) Supported by its **strong** disciplines, the University has built a science and technology industry system which consists mainly of biology and biochemistry, special materials, fine chemicals, electronic information, and mechanical and electrical equipment (Nanjing University, 1997).
(8) SJTU enjoys an increasingly **high** scientific research level and technological innovation level (Shanghai Jiao Tong University, 2021).

#### Faculty Capacity

The academic and research advantages of universities, however, were not always positively evaluated as non-human entities, because all the academic and research outputs were achieved by humans. Advantages in this regard were also appraised from the perspective of human agents or producers. As shown in Extracts 9 and 10, faculties members were frequently praised in terms of their teaching and research abilities.

(9) SJTU’s faculty is, by any measures one might use, one of **the most distinguished** in the nation (Shanghai Jiao Tong University, 1997).
(10) In the 1920s and 1930s, Nankai had already gathered **esteemed** scholars as teachers (Nankai University, 2021).

#### Alumni and Students

In the *About Us* texts, students and alumni were positively appraised as beneficiaries of educational services provided by the universities, as shown in Extract 11. They were often praised as capable actors making contributions to the industry or the entire nation. Extract 12 is a case in point.

(11) Students of CUN enjoy a **colourful and lively life** in the campus (Minzu University of China, 2001).
(12) Since Renmin University of China was established, more than 250,000 **outstanding** RUC alumni have made important contributions in various sectors including government, academia, finance, communication, and education (Renmin University of China, 2021).

#### International Collaboration and Cooperation

Internationalisation is another selling point for the top-tier universities. The prestigious universities have more access to international cooperation, such as academic exchange programms and research collaboration, than other universities do. These activities were often underscored by the elite universities to attract students who are interested in such international opportunities to broaden their horizons. Extracts 13 and 14 are examples where international collaboration and cooperation are positively evaluated.

(13) Xiamen University has promoted **extensive** international academic exchanges, and has the capability of academic exchanges in all subjects and with all countries and regions (Xiamen University, 1996).
(14) As one of the first Chinese universities to carry out international Chinese education, it enjoys a **leading** position in this field (East China Normal University, 2021).

### Diachronic Differences in the Use of Attitude Markers

Although the *About Us* texts produced at both time points used positive Attitude markers to highlight the seven major themes summarised above for the promotional purpose, quantitative and qualitative analyses did reveal some differences in the use of Attitude markers between the two time points. The following section reports the identified diachronic differences.

As mentioned earlier, four Wilcoxon signed-rank tests were run to compare the relative frequencies of attitude markers in the *About Us* texts at the two time points. The Wilcoxon signed-rank test on Attitude as an aggregate category showed that there was a significant difference between the two corpora. The texts in Phase II (*M* = 52.64, SD = 2.99) used significantly more Attitude markers than those in Phase I (*M* = 39.43, SD = 3.20), *Z* = −2.817, *p* = 0.005. Under this aggregate Attitude category are subcategories of Affect, Judgement, and Appreciation.

#### Affect

The Wilcoxon signed-rank test on Affect indicated that the two corpora used this sub-type of Attitude markers with similar frequencies, *Z* = −0.051, *p* = 0.959. Affect markers appeared with very low frequencies in both Phase I corpus (*M* = 0.34, SD = 0.17) and Phase II corpus (*M* = 0.29, SD = 0.12). The universities revealed positive feelings and emotions expressed by both faculty and students. Notably, their happiness was most frequently associated with the universities’ services and facilities, as demonstrated in Extracts 15 and 16.

(15) All nationalities keep their good traditional customs and **celebrate** their holidays (Minzu University of China, 2001).
(16) Its students fully **enjoy** global high-quality educational resources (Beihang University, 2021).

However, a close examination of the Affect extracts from the two corpora revealed three differences in the use of this sub-type of Attitude markers. First, the Phase II (2021) corpus contained more instances that positively appraised students than did the Phase I (turn-of-the-century) corpus. Second, the Phase II corpus showed a more diverse use of Affect markers than the other corpus did. For example, aside from Happiness manifested by the Extracts 15 and 16, instances of Desideration and Satisfaction were found in the Phase II corpus. The bolded segment in Extract 17 (i.e., *eager to*) expresses a feeling of eagerness, and that in Extract 18 (i.e., *take pride*) indicates satisfaction. Third, informal features were observed in the 2021 corpus. For example, the use of personal pronouns such as *we*, *you*, *your*, *us* in Extract 19 helps to construe a conversational tune, which suggests an attempt to talk directly with readers of the texts and to establish a close relation with stakeholders of the universities.

(17) The goal of talent cultivation is to nurture students who have both ability and moral integrity, able to cultivate charisma and **eager to** serve their country (Sun Yat-sen University, 2021).
(18) With the development strategy to “pillar our university with academic achievements, empower it with talents, make it prosper through innovation, enliven it with an open atmosphere, and **take pride** in its unique culture”, our university aims to become a high-level research university (Jilin University, 2021).
(19) We **welcome** your cooperation in meeting the challenges of an ever-changing world and invite you to join us in building a world-class university and promoting excellence in higher education (Renmin University of China, 2021).

#### Judgement

The test on Judgement also showed a non-significant difference between Phase I (*M* = 7.57, SD = 1.06) and Phase II corpora (*M* = 11.28, SD = 1.34), *Z* = −0.149, *p* = 0.139. Judgement markers were used in both corpora to highlight faculty’s and students’ academic and research capacities (Extract 20), tenacity in their pursuit of academic excellence (Extract 21), and commitment to virtue ethics (Extract 22).

(20) MUC has always had **high-quality** faculty (Minzu University of China, 2021).
(21) The staff members are **making every endeavour** to build the university into a highly advanced institution of educational science and other sciences (East China Normal University, 1997).
(22) Many others are **well-qualified** in both academic and **ethical aspects**, making **remarkable contributions** to the construction of our socialist modernisation (University of Electronic Science and Technology of China, 1998).

It should be noted that when people’s virtue ethics or moral standards were evaluated in the texts, as illustrated in the last extract, they were often associated with patriotism and ideological perspectives. In other words, political correctness and loyalty to the Communist Party of China (CPC) and the Socialist cause are considered indispensable to the virtue ethics in the Chinese higher education context.

#### Appreciation

Finally, the Wilcoxon signed-rank test on Appreciation indicated a significant difference between the two corpora, *Z* = −2.113, *p* = 0.035. The *About Us* texts posted in 2021 (*M* = 41.07, SD = 2.97) used significantly more Appreciation markers than the texts posted at the turn of the century (*M* = 0.31.52, SD = 2.91). As shown in Table 2, Appreciation markers, which are principally concerned with the evaluation of tangible non-human entities and intangible ideas, took up the highest proportion of all Attitude markers. The universities often used them to foreground their internal or external environment (Extract 23), social status (Extract 24), the achievements they made (Extract 25), and the abstract qualities they possess (Extract 26).

(23) Backed by the **majestic** Yuelu Mountain and facing the **grand** Xiangjiang River, CSU has **pleasing** scenery and is **ideal** for study and research (Central South University, 2021).
(24) 40 years’ development has shaped East China Normal University into a **prestigious** teacher training institution, influential both at home and abroad (East China Normal University, 1997).
(25) CSU has made **great progress** in its overall strength through teamwork, innovation and commitment to excellence (Central South University, 2021).
(26) The **outstanding** tradition dates back to the early 20th century, when modern democracy and science were emerging and developing quickly in Guangdong province (South China University of Technology, 2021).

There seemed to be a growing number of Appreciation markers that positively evaluated the external and internal environment in the texts over the past 20 years. Such markers, though appraising things and entities, are related to human perception and can invoke people’s Affect. The increase in their frequency in the Phase II corpus indicates more human involvement in the creation of the *About Us* texts in 2021.

### Discursive Marketisation Through Positive Evaluation

From both the major themes evaluated and the diachronic difference over the past two decades summarised above, it is not difficult to realise that positive evaluation has been strategically employed by the top-tier Chinese universities to extensively conduct self-promotion, to aggressively attract potential students, and to actively compete in the higher education market.

More specifically, these universities like market vendors are explicitly promoting what they have to offer, such as educational resources, facilities, environments, products and services, to prospective students who, like consumers in the market, have free choices and decisions to make. Over the past 20 years or so, more and more Attitude markers were used in the *About Us* texts, more students-related evaluations were deployed, more diverse and informal Attitude instances were utilised, and more Attitude instances were employed to boast a cosy and comfortable environment that students could enjoy in and outside of the campus. All these discursive strategies jointly pointed to a clear tactic on the part of the universities: to gain the students’ (i.e., the consumers’) favour and to influence their choices and decisions. This, in part, reflects an ongoing transition to marketisation within the context of Chinese higher education over the two decades.

## Discussion

### Promoting Chinese Higher Education: The Influence of Market, Government, and Tradition

The findings summarised in the previous section can be explained by a host of factors exerting influence on Chinese higher education. One prominent factor is related to the marketisation of higher education, which has been intensified by the trend of globalisation. The market-oriented economy in China has replaced the old-fashioned plan-oriented economy and the market-oriented system has been gradually introduced in the domain of higher education. One significant change in Chinese higher education lies in the shift from elite higher education to mass higher education (Hayhoe, 2012; Mok and Jiang, 2016). The massification of higher education has rendered students more choices. As a result, the Chinese universities are taking measures to enhance “consumer choice” in the “quasi-markets” (Mok, 2000, p. 111). This partly accounts for the more human-related, and especially student-related, Attitude markers in the *About Us* texts in the Phase II corpus. Relatedly, the presence of conversational and informal style in the Phase II corpus could be attributed to the fact that the top-tier Chinese universities are pulling down their poker faces and vying for quality students in the competitive higher education market. The marketisation logic also endows universities with relatively higher institutional autonomy as compared to decades ago (Pan, 2009), though Chinese higher education is still known for its culture of uniformity and conformity (Cheng, 2006).

The second factor is concerned with higher education policy, more specifically, the way in which the universities are governed. Different from their Western counterparts that have seen a reduced role of government due to the influence of neoliberalism, Chinese universities have witnessed a persistently central role played by the state. The governance of Chinese higher education is unique and is emerging as the Chinese model (Marginson, 2011; Li, 2012). Underlying the governance are two fundamental functions to be performed by the universities: transmitting knowledge for the purpose of national rejuvenation and transmitting to students a set of socio-political values prescribed by the state (Pan, 2009). For the first function, facilitated by state initiatives, China’s economic modernisation is expected to advance the development of selected elite universities. In terms of the second function, patriotic sentiment and the red revolutionary spirit are constantly emphasised in the cultivation of talents devoted to the Socialist cause. As rightly pointed out by Saichaie and Morphew (2014), university websites can reveal the purpose of higher education followed by the institutions. Patriotism and embracement of the dominant ideology can be easily traced in the *About Us* corpus. Extracts 27 and 28 are instances where contribution to the motherland is clearly articulated.

(27) They all play important roles in their own fields and **contribute significantly to the nation construction and development** (Renmin University, 1999).
(28) In the past 58 years since its founding, BIT has turned out more than 60,000 scientists and technologists **for the country** (Beijing Institute of Technology, 1999).

The negative Attitude markers provide an interesting perspective on this point. Both Extracts 29 and 30 are instances of negative evaluation. However, these negative Attitude markers are not directed at the universities but at the foreign invaders in the first extract and at the historical situation of the nation in the second. The negative markers in the extracts function to arouse a sentiment of patriotism, which seldom fails to evoke solidarity and empathy among the Chinese readers.

(29) In 1937, the University experienced **devastating damage** at the hands of the invading Japanese army (Nankai University, 1998).
(30) Nankai University (NKU) was founded in 1919, by the famous patriotic educators Zhang Boling and Yan Xiu, when the thunder of the May Fourth Movement was just about to explode and the whole China had fallen into **a life–and–death crisis** (Nankai University, 2021).

It should be acknowledged, however, that little is known about international students’ views on such ideology and revolutionary spirit. Given that the English version of the *About Us* texts targets primarily at international readership, these Chinese universities need to examine the effectiveness of this promotional strategy in attracting international prospective students. In our comparison of the Chinese and English versions of the *About Us* texts of the same universities, it was clear that the English versions of some universities’ *About Us* texts were translated word-for-word from the Chinese versions. Considering the fact that what works for attracting Chinese students may not necessarily work for attracting international students, it stands to reason that Chinese universities should tailor their *About Us* texts specifically to international prospective students so as to better suit their reading habits and expectations as consumers of higher education.

Another factor is associated with Confucianism which has long penetrated into the education sphere in China. The Confucian doctrine stresses five integral virtues of man: benevolence (仁), righteousness (义), trustworthiness (信), propriety (礼), and wisdom (智) (Waley, 2012). The thinking has such an enormous impact on current Chinese education that “establishing moral integrity and cultivating talents” (立德树人) has become the fundamental task in higher education nationwide. It could therefore partly account for the considerable number of Attitude markers that positively evaluate the moral aspect of faculty and students in the *About Us* corpus.

### Shaping Student Choice: The Role of Social Cognition

It is noteworthy to mention the psychological mechanism by which student choice could be influenced by the promotional strategies employed by the top-tier Chinese universities. According to van Dijk’s (2009) Discourse-Cognition-Society triangle model, language use and social interactions can influence each other through people’s “cognitive interface of mental models, knowledge, attitudes and ideologies” (p. 64). The interface involves cognitive processes and mental representations in the comprehension of discourse. In the case of this study, the *About Us* texts are meant to be interpreted by student audience with shared mental models, knowledge, and attitudes situated in a specific epistemic community. In this process, positive Attitude markers predispose the top-tier universities to be perceived by student audience as authoritative, trustworthy, or credible agents, whose beliefs, claims, knowledge, and opinions inscribed in the texts tend to be accepted by the students (van Dijk, 2015). Therefore, when students’ attitudes constructed in the specific epistemic community are aligned with the attitudes construed in the texts, shared social cognition operates to legitimise the institutional promotional activities and thereby shape student choice.

### The Emergence of a Higher Education System With Chinese Characteristics

As discussed above, the marketisation process has influenced the outlook of Chinese higher education, which was once characterised by state planning. However, students now are no longer enrolled according to pre-allocated recruitment quotas, nor are they assigned to pre-defined working positions upon graduation. Features such as “quasi-markets” and “consumer choice” have brought out a higher degree of diversity in Chinese higher education (Mok, 2000, p. 111). This indicates that there are both commonalities and differences between the Chinese higher education system and the Western higher education system.

The differences between the Chinese and the Western higher education systems, in particular, are rooted in a series of socio-political imperatives, socio-economic constraints and socio-cultural norms that are in constant dialogue with each other (Xie and Teo, 2020a,b). While government authority headed by the MOE predominates in Chinese higher education, market forces exert considerable impact on Western universities, which enjoy much more institutional autonomy (e.g., Fairweather, 2000; Ding and Levin, 2007; Pan, 2009). In exploring the diversification/homogenisation in American universities, Fairweather (2000) published an article entitled *Diversification or homogenisation: How markets and governments combine to shape American higher education*. Almost a decade later, Zha’s (2009) article addressed a similar issue in the Chinese context: *Diversification or homogenisation: How governments and markets have combined to (re) shape Chinese higher education in its recent massification process*. The reverse order of “governments” and “markets” in the two article titles is, in a sense, indicative of the relative weighting of the two major driving forces that shape modern higher education in their respective socio-political contexts. Correspondingly, the motivation to serve the national interest seems to outweigh the aspiration to satisfy students’ personal needs in the Chinese higher education system. The opposite seems to be true in the Western context, where investment-return logic is celebrated and personal development tends to be prioritised over other social agendas.

This difference is related to another observation clearly seen in the extracts presented earlier: Chinese graduates are encouraged by the universities to serve their country and take pride in their traditional culture. In contrast, many Western universities endorse the individualistic spirit and encourage students to challenge orthodoxy and bring about change. This difference can be attributed to the collectivism-individualism distinction between China and the West (Hofstede, 2001). China is recognised as a typical collectivist culture characterised by a preference for group identification and interconnectedness among members within a largely homogeneous cultural community. By contrast, the Western world often valorises individual autonomy, which places emphasis on individuals’ needs and goals, and prefers looser ties among social members (e.g., Xu and Xia, 2013; Chen, 2017).

Mirroring market economy with Chinese characteristics, we can come up with a term to capture the ground reality in the domain of higher education in China: higher education with Chinese characteristics. In this emerging model, while the effect of neoliberalism has been tempered and that of the marketisation logic mediated, the role of the government has been administratively effective and financially supportive.

## Conclusion

This study investigated the marketisation process of Chinese higher education by comparing the use of Attitude markers in the *About Us* texts posted on the official websites of 35 top-tier Chinese universities at the turn of the century and in the year 2021. A high density of positive Attitude markers revealed the promotional nature of the texts and the seven major themes positively appraised in the texts during the past two decades, which reflects in part the discursive strategies employed by top-tier Chinese universities to attract prospective students and engage in competition in the higher education market.

Quantitative and qualitative analysis of the texts also showed some mild changes in the use of the markers during the timeframe. The analysis of the Attitude markers uncovered that the strategic promotion of China’s top universities seems to be positioned in a constant paradox between marketisation paralleling that in the Western world and government guidance and support unique in China. The equilibrium between the two forces appears to have generated some motives for the progress made by the top-tier Chinese universities so far. The confrontation and interaction of the two are further compounded by the patriotism and traditional Chinese values most typically represented by Confucianism. As an idiosyncratic model, Chinese higher education, as well as the way it is promulgated, deserves more scholarly attention.

This study can offer some implications for examining the discoursal construction for institutional promotional purposes. First, as demonstrated in this study, attitude markers constitute an integral part of the self-evaluation and self-promotion process. By focussing on the attitude dimension in given texts, the discursive strategies for constructing institutional images and university-reader relationships can be unveiled. Second, this study adopted a mixed method research design, where both detailed analysis of textual features and statistical procedures were performed to explore the discursive strategies utilised by the universities. The integration of both research paradigms can be expected to yield a more holistic understanding of the working mechanism where linguistic features constitute, and at the same time are shaped by, the social reality, in this case the Chinese higher education. One possible option for future research in this line of inquiry might be an empirical investigation into the similarities and differences between public and private universities in discursively forging their institutional images and establishing university-audience relationships.

## Data Availability Statement

The raw data supporting the conclusions of this article will be made available by the authors, without undue reservation.

## Author Contributions

CX and JL conceived the idea and decided on the research design. LX collected and analysed the data. CX and LX drafted the manuscript. JL revised, proofread, and finalised the manuscript for submission. All authors contributed to the article and approved the submitted version.

## Conflict of Interest

The authors declare that the research was conducted in the absence of any commercial or financial relationships that could be construed as a potential conflict of interest.

## Publisher’s Note

All claims expressed in this article are solely those of the authors and do not necessarily represent those of their affiliated organizations, or those of the publisher, the editors and the reviewers. Any product that may be evaluated in this article, or claim that may be made by its manufacturer, is not guaranteed or endorsed by the publisher.

## Footnotes

1
http://wayback.archive.org/
